# Dermatomyositis in a young patient: A rare paraneoplastic syndrome of renal cell carcinoma

**DOI:** 10.1002/iju5.12754

**Published:** 2024-07-15

**Authors:** Margarida André, Alexandre Macedo, Vanessa Metrogos, Luísa Moreira, José Pereira, Nuno Figueira, João Paulo Rosa, Miguel Carvalho

**Affiliations:** ^1^ Urology Department Hospital Garcia de Orta EPE Almada Portugal; ^2^ Urology Department Instituto Português de Oncologia de Lisboa Lisbon Portugal; ^3^ Radiology Department Hospital Garcia de Orta EPE Almada Portugal

**Keywords:** dermatomyositis, paraneoplastic syndrome, renal cell carcinoma

## Abstract

**Introduction:**

Paraneoplastic syndromes are frequent in patients with renal cell carcinoma. Dermatomyositis is an idiopathic inflammatory myopathy that may be associated with neoplasms. This case aims to describe the rare association of dermatomyositis with renal cell carcinoma and to increase clinical suspicion of this neoplasm when systemic rheumatologic symptoms are present.

**Case presentation:**

A 35‐year‐old female developed severe rheumatologic symptoms (progressive muscle weakness, heliotrope rash, and Gottron's papules). Clinical and laboratory findings indicated dermatomyositis. During the investigation, computed tomography revealed a left kidney solid mass. She underwent a left radical nephrectomy, and histology confirmed renal cell carcinoma. After 3 months, dermatomyositis manifestations disappeared and the patient withdrawn steroid therapy.

**Conclusion:**

Most paraneoplastic syndromes associated with renal cell carcinoma are only cured with nephrectomy. We highlight the potential role of surgery in dermatomyositis caused by renal cell carcinoma. The recurrence of symptoms related to the syndrome should alert for disease progression.

**Abbreviations & Acronyms**
AFP: alpha‐fetoprotein
ANA: antinuclear antibodies
CK: creatine kinase
CT: computed tomography
DM: dermatomyositis
EMG: Eletromyogram
ENA: extractable nuclear antigens
IIM: idiopathic inflammatory myopathy
MRI: magnetic resonance imaging
RCC: renal cell carcinoma

**Keynote message**

Dermatomyositis is an idiopathic inflammatory myopathy that sometimes presents as a paraneoplastic syndrome but is rarely associated with renal cell carcinoma. This clinical case intends to raise awareness of this association.

## Introduction

Renal cell carcinoma (RCC) is the third most frequent genitourinary cancer worldwide. Nowadays, <15% of patients present with the classic diagnostic triad of palpable mass, flank pain, and hematuria.

Paraneoplastic syndromes are defined as a set of signs and symptoms, secondary to a malignancy, but excluding the direct effect of tumor extension or metastasis. Regarding RCC, it is estimated that 10% to 40% of patients will develop a paraneoplastic syndrome at diagnoses.^1^

Dermatomyositis (DM) is an infrequent idiopathic inflammatory myopathy (IIM) that affects predominantly the proximal skeletal muscles and skin. Its incidence is estimated at 9.63 per 1 million people.^2^ There is a higher prevalence in the mid‐50 to 60 years old, being around two times more frequent in females.^3^ DM is associated with malignancies, and in some rare cases, it can be the first clinical manifestation. Breast, lung, ovarian, and colorectal cancer are commonly reported associations with DM.^4^ However, there is a paucity of data regarding RCC and DM, especially the occurrence of the disease in young patients.

## Clinical case

**Fig. 1:**
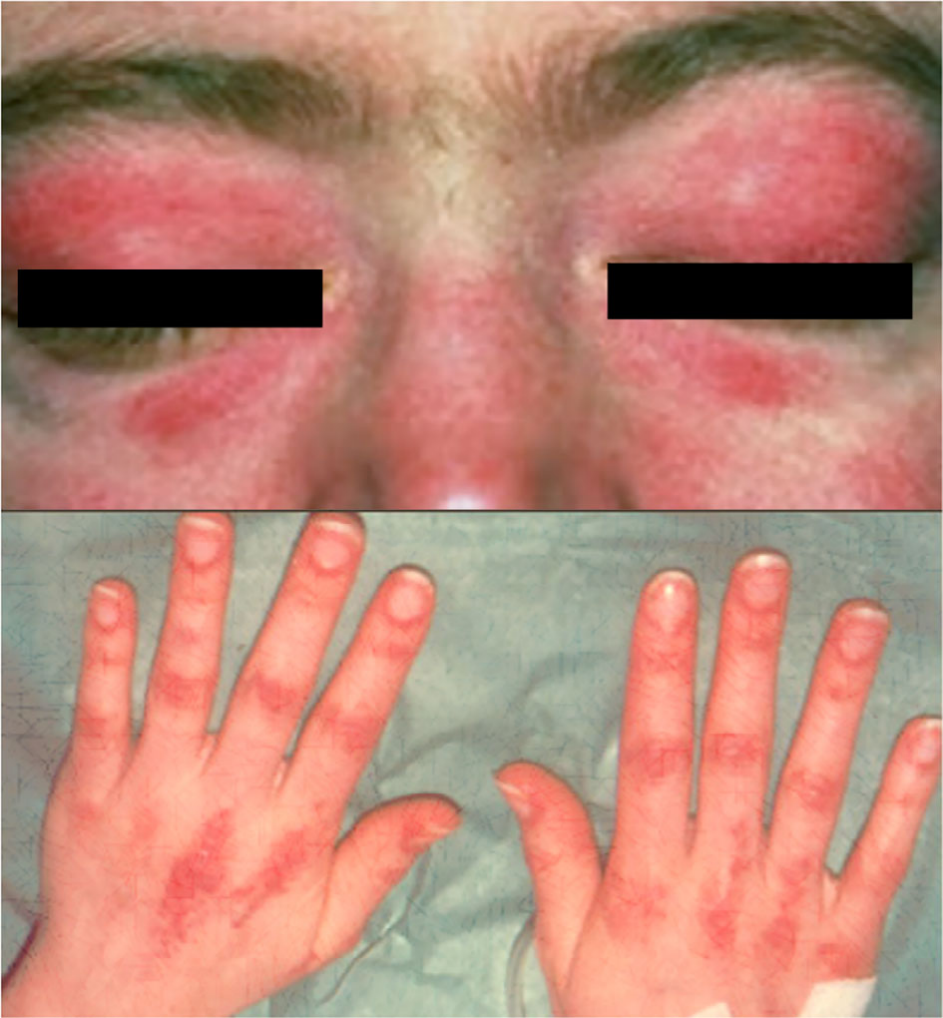
Heliotrope rash and Gottron's papules.

A 35‐year‐old Caucasian female, with a known medical history of hypothyroidism due to auto‐immune thyroiditis and ex‐smoker (20 pack‐years), presented with a 3‐month history of symmetrical progressive muscle weakness and cutaneous rash in the face and hands. She works as a cook and has had no exposure to radiation or industrial chemicals. Physical examination revealed proximal muscle weakness, heliotrope rash (edematous erythema of the eyelids), and Gottron's papules (erythematous papules on the dorsal surface of the phalangeal joints) (Fig. 1). Laboratory evaluation revealed an elevated creatine kinase (CK) of >2000UI/L (normal range 0–70 U/L), myoglobin 491 ng/mL (normal range < 90 ng/mL), lactate dehydrogenase 718 UI/L (normal range 120–246 UI/L ng/mL), and negative antinuclear antibodies (ANA). Other routine tests, including anti‐ENA (extractable nuclear antigens), protein electrophoresis, and urinalysis, were normal. Tumor markers CA 19‐9, CA 125, CA 15‐3, CEA, and alpha‐fetoprotein (AFP) were also unremarkable.

Eletromyogram (EMG) of the affected muscles revealed muscle weakness. Magnetic resonance imaging (MRI) did not reveal pathologic findings. Muscle biopsy was not performed.

According to these findings, dermatomyositis was diagnosed based on Bohan and Peter criteria, with four out of five criteria confirming a “definitive” diagnosis (proximal symmetrical muscle weakness, elevation of serum skeletal enzymes, EMG findings, and heliotrope rash).^5^, ^6^

Treatment was started with subcutaneous methotrexate and oral prednisone with only mild relief of the symptoms. Three months after initiating treatment, the patient was admitted to the Rheumatology Department for Investigation.

**Fig. 2:**
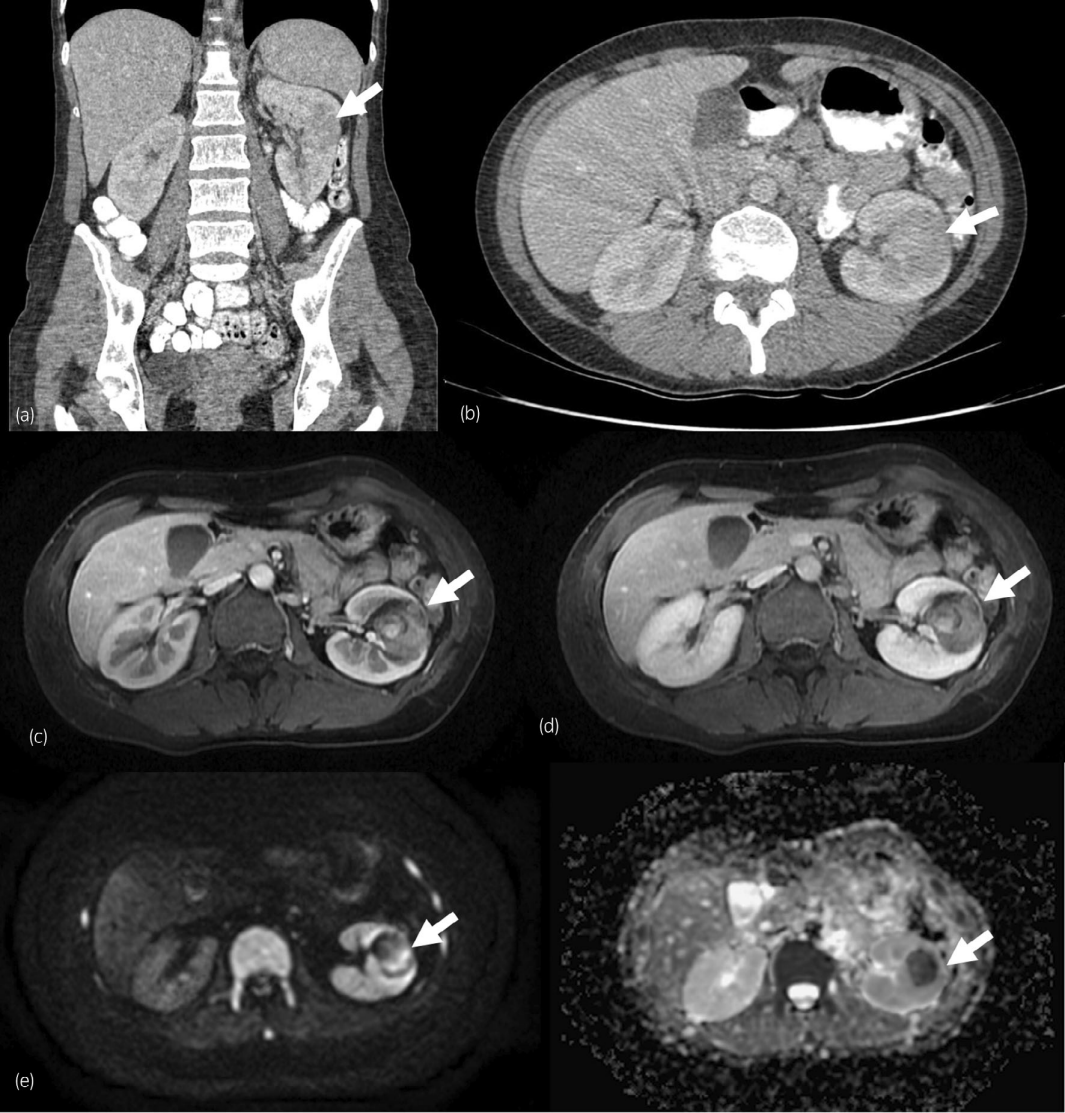
Solid mass on the left kidney. (a) CT portal venous phase, coronal image; (b) CT portal venous phase, axial image; (c) MRI arterial phase, axial image; (d) MRI venous phase, axial image; (e) MRI DWI/ADC showing diffusion restriction of the lesion, axial image.

The possibility of malignancy associated with dermatomyositis as a paraneoplastic syndrome was evaluated. A diagnostic chest, abdomen, and pelvic computed tomography (CT) revealed a 35 × 32 × 28 mm left kidney mass, with contrast enhancement, suggestive of RCC (Fig. 2). An abdominal MRI (Fig. 2) was performed to exclude the presence of lymphoma. FDG‐PET excluded the presence of other malignant pathologies.

**Fig. 3:**
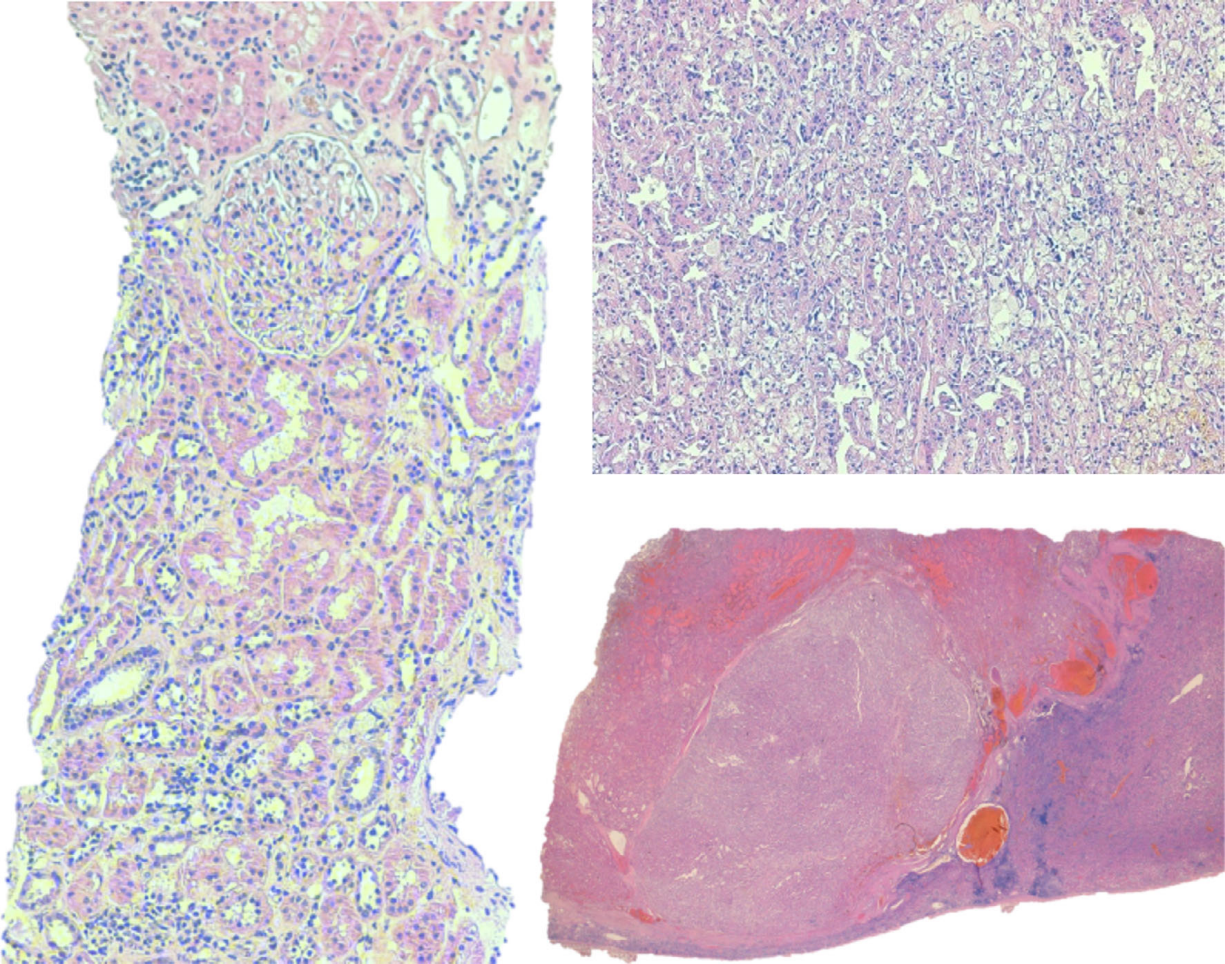
Histology of radical left nephrectomy sample was compatible with renal cell carcinoma (clear cell, Fuhrman 3, pT1a).

The patient was admitted to the Urology Department and was submitted to laparoscopic left radical nephrectomy. Histopathology revealed a clear cell carcinoma, with focal nuclear changes graded as Furhman 3, without lymphovascular invasion, confined to the kidney (pT1a) (Fig. 3). Four weeks after nephrectomy (still under corticotherapy), there was a clinical and laboratorial evident improvement, with no identifiable cutaneous rash and CK decreased to <100 U/L. Three months after the surgical procedure, there was remarkable progress in the clinical status with normalization of the muscle strength and CK levels. The patient gradually stopped steroids at this time. Four years after the procedure, the patient remains asymptomatic, with a negative CT scan on follow‐up.

## Discussion

Paraneoplastic syndromes' association with RCC is a relatively common advent in the diagnostic workup of this pathology, such as hypercalcemia, hypertension, hyperglycemia, hypercortisolism, hepatic dysfunction such as Stauffer syndrome, syndrome of inappropriate ADH production and galactorrhea, hematologic syndromes and neuromuscular disorders.^7^ Although the clinical manifestations of paraneoplastic syndromes can present early and precede the diagnosis of RCC, they are usually associated with advanced tumors and metastatic disease.^8^

There is no established association between DM and RCC, and very few case reports have described similar findings (Table 1).^9^, ^10^, ^11^, ^12^, ^13^, ^14^

**Table 1:** Clinical characteristics of the present case and previous published clinical cases

Author, Year	Age, sex	Clinical presentation	Renal malignancy	Management	Histopathology
Present case	35, female	Proximal muscle weakness, heliotrope rash, and Gottron's papules	3.5 cm left renal mass	Laparoscopic left radical nephrectomy	pT1a, Clear cell RCC, with focal nuclear changes, Fuhrman grade 3
Srivastava *et al*., 2022^9^	43, male	Proximal muscle weakness, myalgia, and maculopapular rash over the upper chest and back	2 cm right renal mass	Laparoscopic partial nephrectomy	RCC
Kyaw *et al*., 2017^10^	72, male	Generalized weakness and dysphagia	Left renal mass	Radiology–guided renal artery chemoembolization	Clear cell RCC
George *et al*., 2016^11^	27, male	Proximal muscle weakness, myalgia, dysphagia, and cuticular and periungual erythema	2 cm right renal mass	Partial nephrectomy	pT1a, Chromophobe RCC, Fuhrman grade 3
Adili *et al*., 2015^12^	69, male	Grotton's papules	5.8 cm left renal mass	Radical nephrectomy	Clear cell RCC, Fuhrman grade 3/4
Nevins *et al*., 2013^13^	77, female	Gottron's papules, heliotrope rash, and proximal muscle weakness	4 cm left renal mass	Radical nephrectomy	Clear cell RCC, Fuhrman grade 2
Schaefer *et al*., 2004^14^	71, female	Fatigue and muscle weakness, poikiloderma, dysphagia, cardiopulmonary dysfunction	6 cm left renal mass	Arterial embolization and CT‐guided percutaneous Radiofrequency Ablation	RCC

DM has been associated with malignant neoplasms since 1916, when Stertz reported the first two cases of IIM related to stomach cancer.^15^ Evidence supporting this association has shown an incidence of 7%–30%; however, RCC was not found in this population set.^15^, ^16^ Even though previous reports (highlighted in Table 1) may suggest that DM can present in relatively early‐stage RCC, but more studies are necessary to correlate this paraneoplastic syndrome with RCC histology, grade, and stage. Cancer diagnoses usually occur in the year after the diagnosis of DM. Several risk factors take part for this condition including age over 60, male gender, diabetes, dysphagia, skin necrosis, Gottron's sign, an onset inferior to 4 weeks, elevated CK, erythrocyte sedimentation rate (ESR), C‐reactive protein, and CA‐125.^17^, ^18^

The underlying mechanism for the development of cancer in IIM is still not well established. The theory of cancer auto‐immunity has been consistently proven to play a role in this association. Many myositis‐specific autoantibodies (MSA), such as anti‐TIF1γ, were detected in large cohort studies as the origin of carcinogenesis, which can lead us to elevate the clinical suspicion of neoplasia and advocate screening of these patients.^18^, ^19^ Detection of MSA's is not widely performed due to its unavailability and high cost.

Selecting these patients is a subject of debate, but it should be based on the risk factors previously described or positive autoantibodies. Evaluation should take into consideration a complete blood count, physical examination including gynecological exam, tumor markers (CA‐125, CA 19‐9, AFP), mammography and breast ultrasound, and a thoraco‐abdomino‐pelvic CT scan.

The role of tumor excision is well established as a treatment for this syndrome, as we saw in our case with regression of all symptoms after surgery. Vigilance is crucial, as the risk for malignancy remains high even 1 year after the diagnosis.^20^

This case illustrates the extreme value of a careful clinical history, physical examination, and complementary evaluation for the diagnosis of malignancy in patients with DM. Although rare, we highlight the association RCC‐DM, even in young patients.

## Author contributions

Margarida André: Writing – original draft; writing – review and editing. Alexandre Macedo: Writing – review and editing. Vanessa Metrogos: Writing – review and editing. Luísa Moreira: Writing – review and editing. José Pereira: Resources; writing – review and editing. Nuno Figueira: Writing – review and editing. João Paulo Rosa: Writing – review and editing. Miguel Carvalho: Writing – review and editing.

## Conflict of interest

The authors declare no conflict of interest.

## Approval of the research protocol by an Institutional Reviewer Board

Not applicable.

## Informed consent

Not applicable.

## Registry and the Registration No. of the study/trial

Not applicable.
